# Consistency and accuracy of diagnostic cancer codes generated by automated registration: comparison with manual registration

**DOI:** 10.1186/1478-7954-4-10

**Published:** 2006-09-28

**Authors:** Giovanna Tagliabue, Anna Maghini, Sabrina Fabiano, Andrea Tittarelli, Emanuela Frassoldi, Enrica Costa, Silvia Nobile, Tiziana Codazzi, Paolo Crosignani, Roberto Tessandori, Paolo Contiero

**Affiliations:** 1 Cancer Registry Division, Istituto Nazionale per lo Studio e la Cura dei Tumori, Via Venezian 1, 20133 Milan, Italy; 2 Province of Sondrio Health Authority, Via Stelvio 35A, 23100, Sondrio, Italy

## Abstract

**Background:**

Automated procedures are increasingly used in cancer registration, and it is important that the data produced are systematically checked for consistency and accuracy. We evaluated an automated procedure for cancer registration adopted by the Lombardy Cancer Registry in 1997, comparing automatically-generated diagnostic codes with those produced manually over one year (1997).

**Methods:**

The automatically generated cancer cases were produced by Open Registry algorithms. For manual registration, trained staff consulted clinical records, pathology reports and death certificates. The social security code, present and checked in both databases in all cases, was used to match the files in the automatic and manual databases. The cancer cases generated by the two methods were compared by manual revision.

**Results:**

The automated procedure generated 5027 cases: 2959 (59%) were accepted automatically and 2068 (41%) were flagged for manual checking. Among the cases accepted automatically, discrepancies in data items (surname, first name, sex and date of birth) constituted 8.5% of cases, and discrepancies in the first three digits of the ICD-9 code constituted 1.6%. Among flagged cases, cancers of female genital tract, hematopoietic system, metastatic and ill-defined sites, and oropharynx predominated. The usual reasons were use of specific vs. generic codes, presence of multiple primaries, and use of extranodal vs. nodal codes for lymphomas. The percentage of automatically accepted cases ranged from 83% for breast and thyroid cancers to 13% for metastatic and ill-defined cancer sites.

**Conclusion:**

Since 59% of cases were accepted automatically and contained relatively few, mostly trivial discrepancies, the automatic procedure is efficient for routine case generation effectively cutting the workload required for routine case checking by this amount. Among cases not accepted automatically, discrepancies were mainly due to variations in coding practice.

## Background

The data provided by cancer registries are vital for public health surveillance, health service planning, evaluation of the impact of interventions on cancer incidence and survival, clinical auditing, and epidemiological research. They also provide information useful for health promotion and genetic counseling. To fulfill these functions adequately, the data produced by cancer registries must be of high quality [1]. Incomplete and inaccurate data registration may introduce serious bias to estimates of cancer survival [2,3] and cancer incidence rates [4-6].

Advances in information technology have influenced all aspects of health care. In the field of cancer registration, electronic data transfer reduces transcription errors, reduces the need for manual data extraction, promises to reduce the time required to publish and analyze data, and is generally more cost-effective than traditional manual methods. It has thus become feasible to generate cancer incidence data automatically from electronically transferred data using computer programs, with further savings of time and manpower. It is important, however, to verify the accuracy and consistency of the automatically-produced data.

In the present study we have validated automated registration by comparison with manual registration for an entire incidence year. The first advantage of this comparison method is that it evaluates the entire automated registration procedure, including source acquisition, record linkage and algorithm function. Each of these procedures may be associated with inaccuracies which influence the overall accuracy of the automated process. The second advantage of the method is that it avoids bias due case sampling (or re-abstraction) as it considers virtually all incident cases in the year.

In 1997 the Lombardy Cancer Registry (LCR), Province of Varese, northern Italy, introduced a computerized automated procedure (Open Registry) to generate cancer incidence data from electronically transferred source files. In the present study, we examine the consistency and accuracy of the automatically generated data by comparison with the manually generated cancer incidence data for the same Varese population in 1997.

## Methods

### Manual registration

Data sources for manual registration were clinical records, pathology reports and death certificates. Trained staff consulted clinical records and pathology reports in the archives of the hospitals and pathology laboratories of the Province of Varese and in selected institutes of neighboring Provinces, abstracting the data onto case forms. Printed death certificates arrived directly from the local health authority.

The items abstracted were demographic data, tumor site, date of diagnosis, nature of diagnostic confirmation, and tumor type according to the International Classification of Disease (ICD-9, WHO 1975, for topography) and International Classification of Disease for Oncology (ICDO-2, WHO 1990, for morphology). Where possible, death certificate only (DCO) cases were validated by back-tracing clinical records and pathology reports. Registrations were routinely checked for consistency by medical staff.

The LCR is tumor-based: multiple primaries occurring in a patient are recorded separately if they differ morphologically (ICDO-2) or occur at different anatomical sites (third digit of ICD-9 site code). In 1976–1997, 84% of cases were verified microscopically. The percentage of DCO cases is on average 2% of the new cancer cases registered in any year [9-11]. These indicators suggest that the LCR data are of acceptable quality.

### Automatic registration

The data sources are hospital discharge files and death certificates (both containing ICD-9 codes) and pathology reports, containing SNOMED codes (version 2, 1979) [12]. These records were designed, and are collected, for purposes other than cancer registration. Electronic files containing these data are sent periodically to the LCR. The Open Registry software translates the SNOMED codes to ICD-9 (1975) codes by using a translation system developed in-house and incorporated into the Open Registry software. Open Registry then links the records of the sources files to aggregate information for person. This is done using deterministic and probabilistic methods [13]. Finally data consistency checks are performed, again by ad-hoc routines within Open Registry.

The incidence data are then generated by Open Registry from algorithms acting on the ICD-9 codes now present for each case in each of the source files. The strategy implemented by the algorithms is to choose the best code from the ones that might be available for each case. The incidence data are generated in three steps:

#### Step 1

The following algorithms are applied in sequence:

• Algorithm (a) (concordant code check). This algorithm checks whether the first three digits of the ICD-9 code agree in all source records. If they do, all records for the case are considered fully concordant and the computation stops; if not the algorithm passes the case records to algorithm (b).

• Algorithm (b) (elimination of generic site codes). If the codes are split between site-specific and generic ones, the algorithm attempts to eliminate the generic code (e.g. 146 over 1499) using a hierarchic table developed in-house, linking specific and generic sites, and also using if necessary the information provided by the fourth digit. If after eliminations, all the remaining codes now agree the case is considered concordant and the computation stops; otherwise the algorithm passes the case records to algorithm (c).

• Algorithm (c) (elimination of metastatic and ill-defined site codes). The algorithm eliminates metastatic site codes when compatible generic or specific codes are present (e.g. 1970 over 162). If the remaining codes are now the same the case is considered concordant; otherwise it is tagged as not concordant.

#### Step 2

New case records generated in step 1 are checked against those already in the LCR database to identify cases with and without a previous cancer diagnosis; the former might be multiple cancer cases. For cases without a previous cancer diagnosis, one of three categories is assigned by Open Registry:

• concordant multiple incident

• unique incident

• non-concordant multiple incident.

For cases with a previous cancer diagnosis, Open Registry assigns one of the following categories:

• previous concordant

• previous non-concordant.

Previous concordant cases are those for which all incident codes past and present are concordant.

#### Step 3

Decision on automatic acceptance

The program is set up to automatically accept records assigned as concordant multiple incident and also those with a single instance (i.e. status unique incident) of code 173 (non-melanoma skin cancer) on a pathology report. Records assigned non-concordant multiple incident or unique incident with code other than 173 on the pathology report, are tagged for manual review.

**Figure 1:**
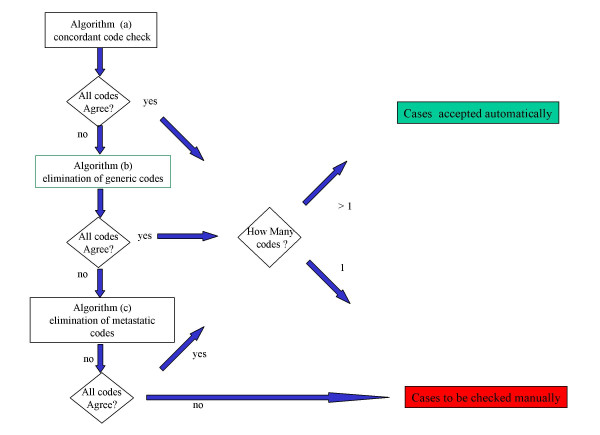
Flowchart of automatic case resolution

A flow chart illustrating the entire procedure is shown in Figure 1. Previous concordant cases do not result in a change in the LCR database. Previous non-concordant cases are reviewed manually.

#### Example 1

A case with 153 (colon cancer) on the pathology report, 153 and 197 (secondary cancer of respiratory and digestive systems) on the hospital discharge form, and 159.9 (cancer of ill-defined site within digestive organs) on the death certificate is passed to algorithm (b) and algorithm (c), which eliminate codes 159.9 and 197, leaving two instances of code 153 (colon). The case is automatically accepted as concordant multiple incident.

#### Example 2

Code 182 (body of uterus cancer) on pathology report of 1997; code 174 (female breast cancer) in LCR database incident in 1995. Such cases are assigned to category previous non-concordant and flagged for manual checking.

### Data comparison

It was found that 2959 (59%) automatically generated cancer cases were accepted by Open Registry because the ICD-9 codes were concordant from all data sources, while 2068 (41%) were flagged for manual verification as the codes were not concordant, or because there was a unique incident code. We carried out comparisons on both the automatically accepted cases (first level comparison) and on the cases flagged for manual checking (second level comparison).

The aim of the first level comparison was to determine the extent of agreement between the automatic and manually produced site codes, and thereby provide an assessment of the quality of the automated case generation procedure.

The second level comparison was carried out to determine the reasons why cases could not be generated automatically: this involved manual comparison of the disease code or codes of each flagged case with the code produced manually. The social security code, present and checked in both databases in all cases, was used to match the files in the automatic and manual databases.

## Results

### First level comparison

#### Identifying and demographic data

Among 2959 automatically accepted cases we had 1.6% surname discrepancies, 2.8% first name discrepancies, 0.07% sex assignment discrepancies, 1.2% date of birth discrepancies, and 2.8% residence code discrepancies (Table 1). The two sex misclassifications almost certainly arose from sexually ambiguous first names. The date of birth discrepancies regarded the year in 6 patients, the month in 7 patients, and the day in 24 patients. All the residence code discrepancies nevertheless placed the case within the Province of Varese. Most of the discrepancies were trivial and none would have disturbed the record linkage performed by the Epilink module [13] of Open Registry.

**Table 1:** Identifying and demographic data discrepancies in automatically accepted cases in comparison to manually generated cases.

**Type of discrepancy**	**No.**	**%**
*Surname*		
Surname spelling	45	1.50
Double surname ambiguity	4	0.14
*First name*		
Spelling of first name	32	1.10
Double first name ambiguity	49	1.70
*Sex*		
Male/female attribution	2	0.07
*Date of birth*		
Year	6	0.20
Month	7	0.24
Day	24	0.81
*Residence code*	84	2.8

#### Site discrepancies

Among the cases accepted automatically, the site code differed from that in the corresponding manually generated incidence case for 1.6% of records. Table 2 categorizes the discrepancies found. Many of the site code discrepancies arose as a result of codification of adjacent sites (e.g. sigmoid colon instead of rectum, biliary system instead of liver) or by codification of a specific primary site instead of metastases or vice versa (e.g. liver metastases instead of primary liver cancer). In seven cases, aspecific hematopoietic system codes were used, whereas the manually-generated case had more specific codes (see Table 2).

**Table 2:** ICD-9 site code discrepancies between automatically accepted cases in comparison to manually generated cases.

**Type of discrepancy**	**No.**	**%**
*Primary neoplasm coded:*		
With different primary site code	28	57
As metastases or unspecified neoplasm	17	35
As neoplasm of uncertain behaviour	1	2
*Ill-defined neoplasm coded as:*		
Primary neoplasm	1	2
Metastases	0	0
*Uncertain behaviour coded as*		
Primary neoplasm	2	4
*TOTAL*	49	100

### Second level comparison

The second level comparison was carried out on the 2068 cases flagged by Open Registry as requiring manual checking. The percentage of automatically accepted cases varied with cancer site (Table 3). Among common malignancies, oropharyngeal cancers (codes 140–149), cancers of non-thyroid endocrine glands (code 194) and cancers of female genital organs (codes 179–184) were characterized by high proportions requiring manual checking. Among the rare cancers, those of the eye (code 190), peritoneum and abdominal cancers, and also ill-defined, metastatic and unspecified site cancers (codes 158–159, 195–199, respectively) had high proportions requiring manual checking. Close to 50% of pleural cancer (code 163), bone and soft tissue cancers (codes 170 and 171), skin melanoma (code 172), and lymphoma (codes 200–202) were accepted automatically.

**Table 3:** Percentages total cases accepted automatically for each site (%).

**Site**	**ICD-9 code**	**Percentage accepted automatically (%)**
Oropharyngeal sites	140–149	25
Digestive tract	150–154	66
	155–157	66
	158,159	22
Respiratory tract	160–161	71
	162	77
	163	50
Bone and soft tissue	170,171	50
Melanoma	172	50
Skin (non-melanoma)	173	77
Breast	174	83
Female genital tract	179–184	40
Prostate	185	77
Male genital tract	186,187	71
Urinary tract	188,189	62
Eye	190	38
Central nervous system	191,192	59
Thyroid gland	193	83
Other Endocrine glands	194	37
Hematological	200–202	43
	203	71
	204–208	50
Other, met. and ill-defined sites	195–199	13

A high percentage indicates that very few cases had to be checked manually.

By contrast, female breast cancer (code 174) and thyroid cancer (code 193) were characterized by high automatic acceptance rate, with only 17% of cases requiring checking.

Table 4 lists the problems found in the flagged cases. Nearly half of the melanoma cases (code 172) had one source code only and for this reason were automatically flagged for manual checking. There was a single data source also for 48.8% of the flagged leukaemia cases (codes 204–208) and 43% of the 14 peritoneal, retroperitoneal and abdominal cancers (codes 158 and 159).

**Table 4:** Problems in cases flagged for checking (selected cancer sites).

**Type of Problem**	No. of cases with specific problems/total flagged cases for cancer site *								
*ICD-9 code*	140–149	158,159	170,171	172	179–184	194	195–199	200–202	204–208
*Cases notified by one source code only*	3/63	6/14	2/13	22/45	32/139	3/7	9/73	20/83	21/43
*Discordant Codes*									
Within a single organ system	25/63	8/14	2/13	0/45	40/139	0/7	16/73	72/83	21/43
Within more than one organ system but not multiple primaries	33/63	0/14	9/13	22/45	65/139	4/7	48/73	3/83	0/43
Multiple primary cancers	33/63	0/14	1/13	1/45	13/139	0/7	0/73	3/83	1/43
Extranodal/nodal site discrepancy	0/63	0/14	0/13	0/45	0/139	0/7	0/73	17/83	0/43

*A case may have more than one problem

One of the most common reasons for flagging was that the codes differed between the various source records. The codes could differ yet with reference to sites within a single organ system (examples: codes 153 and 159 digestive tract cancers; codes 200 and 208 hematopoietic system cancers); or could involve different organ systems (examples: code 200 hematopoietic system and 151 digestive organs; code 170 bone and code 162 respiratory organs).

The presence of different codes referring to a single organ system did not usually indicate a major discrepancy. For example, many oropharyngeal cancer cases had two or more codes (typically 145 and 146) indicating adjacent regions within the oral cavity. Among the discrepant lymphomas, many were due to coding at an extranodal site (e.g. stomach, testis or tonsil; codes 151, 186 and 146 respectively) rather than as a lymphoma (codes 200–202). (Table 4)

When discrepant codes referred to distinct organ systems, the most commonly involved codes were: malignancy of other and ill defined sites (code 195), metastasis (codes 196–198), unspecified site (code 199) and malignancy of female genital tract (codes 179–184).

For example, code 199 often accompanied code 155 (primary liver), but following histological confirmation, the more correct code 197 (metastatic liver disease) replaced codes 155 and 199 in subsequent records (data not shown).

## Discussion

Although the need for quality control of cancer registration data is recognized [14] few data exist on quality of automated cancer registration procedures. Assessments of accuracy using mainly automated methods are available for Arhus County, Denmark [7]; Veneto [8] and Ontario, Canada [7]. The approach in Arhus County was to compare the automatic data with those gathered manually by the Danish national cancer registry. Details of the comparison by site are not available. The approach in Ontario was to re-abstract the data. For Arhus County the reported accuracy level was 98.6% for all cancer sites, and for Ontario the agreement between manual and computer assisted diagnosis was 93.3%. Both these figures are closely comparable to our 98.4%.

In a recently published comparison between manual and automatically generated data for all cancer sites in the Venetian Cancer registry (Italy) [8] concordance was 93.3%. This registry was the first in Europe to use an automatic procedure for cancer incidence generation, and it is reassuring that our concordance level was similar to that obtained by this pioneering approach. The Venetian study used a re-abstraction technique. Although re-abstraction is an excellent method for verifying the accuracy of cancer registration check on a routine basis, in part because it requires limited resources, it is not ideal for evaluating new data collection. We believe that a method that examines all incident cases of a defined time period is more appropriate. We considered a whole year's incidence which provides the opportunity to assess the efficacy of the automated procedure for rare cancer sites.

### First level analysis

#### Identifying and demographic data

Our low rate of surname discrepancies (1.6%) is similar to the 2% found by Doebbeling et al. [15], and the 2.8% discrepancy rate for first names is not too dissimilar to the 4% found in the same study. The study of Brewster et al. in Scotland [16] found a discrepancy of only 0.3% in surnames and first names. Our figure for date of birth discrepancies (1.2%) is lower than reported by Doebbeling et al. (3%) and similar to that of Brewster et al. (1.3 %).

#### Site discrepancies

Our discrepancy rate of 1.6% for automatically accepted cases in comparison with manual registration seems reassuring. Rates reported by other studies have generally been higher (5.4% by Brewster et al. [16], 6% by Lapham et al. [17], 20% by Phekoo et al. [18] in a sample of hematopoietic cancers, and 7% by Dickinson et al. [19]). However, these studies used disparate selection criteria. For example in the study of Phekoo et al. 20% of disagreements concerned morphological coding, so comparison is not straightforward.

Predictably, more than half of our site discrepancies were found, on comparison with manually generated data, to use a generic code instead of a specific code for site, subsite, or histological subtype (the latter for hematologic malignancies). For most of the remaining cases the automatically generated record contained a metastatic code only, obtained from the hospital discharge or pathology files, while the manually-produced data contained a specific site code. This occurred because the manual method involved inspection of the entire clinical record that includes the patient's history and laboratory test results providing information on the site of origin and histology from which the more specific code can be obtained.

### Second level analysis

#### Site discrepancies

In our study the majority of coding discrepancies for site arose through differences in the specificity of coding from one source to another. This could arise, for example in hematologic malignancies, if the disease was initially coded as leukaemia (code 204) on the hospital discharge file, but following histological analysis (reported on the pathology file) it was found to be lymphoma (code 202). For lymphomas, discrepancies also arose because extra-nodal codes were used on one source, while on another source file the more correct generic code was used. Thus, a lymphoma arising in the stomach with characteristics of an extranodal lymphoma could be coded as lymphoma (202) on one source, but stomach cancer (151) on another. Such cases accounted for 20% of the hematological cancers rejected by the automatic system and flagged for manual checking. This revealed an important limit of the automatic system, which cannot be overcome by programming but requires more consistent coding by staff responsible for source record generation. On the other hand, the fact that such cases were blocked by the automatic system shows that it functioned correctly.

Problems of coding hematologic cancers were also raised by Phekoo et al. [18] who compared the diagnostic accuracy of hematologic subtypes obtained from two manual cancer registries, and found that 20% of the examined cases had discordant diagnoses.

A high proportion of oropharyngeal cancer cases were also blocked by our automatic system. The main reason for this was that each specific site code defines a small area that often merges imperceptibly with an adjacent area, so that cancer may be coded for differing, anatomically-close sites, depending on whether the diagnostic approach was clinical or pathological; e.g. tonsillar pillar cancer extending to the palatine plate may be coded as 146 (tonsillar pillars) or 145 (palatine). Such coding difficulties also raise the problem of deciding whether the different site codes indicate the presence of multiple primaries or a single cancer that had extended to other sites. Our approach to addressing this problem when programming Open Registry was to retain information: for two primary sites to be reported in a single individual, the sites had to differ at the level of the third ICD-9 digit and in histology according to the Berg classification [20]. The Berg classification is used by all cancer registries to help ensure that the incidence data they produced are mutually comparable.

The multiple primary versus extended cancer dilemma was particularly evident for cancers of the lip, oral cavity, and pharynx (codes 140–149) where over half of the discrepancies arose for this reason. Middleton et al. [21] have also commented on the difficulties of differentiating on electronic data sources multiple primaries from extended cancers.

We found a relatively high frequency of discrepancies involving cancer from unknown primary site. Hospital discharge files in particular often contained the undefined code 199. In 66% of these cases this code was accompanied by specific site codes, but in all cases manual checking showed that the site codes were for metastatic or disseminated cancers, and it was never possible to identify the site of origin of the primary disease.

In the audit study to assess data quality in the Limberg cancer registry (The Netherlands) [22], it was noted that discrepant cases often involved undefined site codes. The authors proposed as an explanation that 199 was often a preliminary coding, which was later corrected by a more specific code. Another suggested explanation was that the surgeons or clinicians who complete the hospital discharge files had a treatment-centered approach to coding, while registry personnel were interested in precision and natural history.

For genital tract cancers, coding discrepancies were often due to the well-recognized problem of metastases often having different histological characteristics from the primary, and the difficulty of distinguishing metastasis from a second primary. Peritoneal carcinomatosis was often coded as general carcinomatosis (code 199) or malignant peritoneal neoplasm (code 158), and was thus discordant with the primary ovarian (code 183) or uterine (code 182) cancer which gave rise to it. Malignant masses in the abdomen/pelvis were often coded as ill-defined-site within digestive organs and peritoneum (code 159) or as colon or rectal cancer (153 or 154), instead of arising from the female genital tract.

We found that other and ill-defined sites (code 195), peritoneum and ill-defined digestive tract (158 and 159), bone (170) and soft tissue (171) were the sites that most often required manual checking. This is not surprising as these sites give problems for manual registration most often. Similarly, it was reassuring that most other sites were rarely flagged by the automatic system, which was consistent with our manual coding experience that most sites rarely give rise to coding problems.

## Conclusion

In conclusion, the automated method of cancer registration recently introduced for the LCR has been shown to produce good quality data in comparison with the manual method. Since 59% of cases were accepted automatically and contained relatively few and mostly trivial discrepancies, the automatic procedure is efficient for routine case generation effectively cutting the workload required by this amount. Since cancer registries will continue to operate on restricted budgets, it is important that as much registration processing as possible is performed automatically without compromising dataset quality, which thereby frees manpower resources to the more pressing problems of shortening the lag between the acquisition of data and the publication of analyses. It is likely that the feasibility and efficiency of automated registration will improve in the future as health care source data become increasingly available in electronic form.

## Competing interests

The author(s) declare that they have no competing interests.

## Authors' contributions

G. Tagliabue conceived of the study, participated in its design, coordination and analysis, verified diagnoses and clinical standardisations, and drafted the manuscript.

P. Contiero conceived of the study, participated in its design, coordination and analysis, planned data collection and record linkages, and helped draft the manuscript.

A. Maghini, A. Tittarelli, S. Fabiano and T. Codazzi performed automated data acquisition and standardisation, and executed the record linkages.

E. Frassoldi, E. Costa and S. Nobile abstracted clinical information from clinical records.

R. Tessandori and P. Crosignani critically assessed study design and the analysis methods.

All authors read and approved the final manuscript.
